# HIV-related stigma and physical symptoms have a persistent influence on health-related quality of life in Australians with HIV infection

**DOI:** 10.1186/1477-7525-11-56

**Published:** 2013-04-08

**Authors:** Susan Herrmann, Elizabeth McKinnon, Noel B Hyland, Christophe Lalanne, Simon Mallal, David Nolan, Olivier Chassany, Martin Duracinsky

**Affiliations:** 1 Institute for Immunology & Infectious Diseases, Royal Perth Hospital & Murdoch University, Murdoch, Perth, Australia; 2 Sexual Health and Communicable Diseases, Royal Perth Hospital, Perth, Australia; 3 Department of Clinical Research, PRO unit, Assistance Publique–Hopitaux de Paris, Saint-Louis Hospital, Paris, France; 4 Inserm Unit UMR-SO 669, University Paris Sud, Paris Descartes, Paris, France; 5 Department of Clinical Immunology & PathWest Laboratory Medicine, Royal Perth Hospital, Perth, Australia; 6 University Paris-Diderot, Paris, France; 7 Department of Internal Medicine and Infectious Diseases, Le Kremlin-Bicêtre, Assistance Publique–Hopitaux de Paris, Kremlin-Bicêtre Hospital, Paris, France

**Keywords:** HIV/AIDS, Health related quality of life, Health status indicators, Stigma, Symptoms, PROQOL-HIV

## Abstract

**Background:**

The health-related quality of life (HRQL) of people living with HIV infection is an important consideration in HIV management. The PROQOL-HIV psychometric instrument was recently developed internationally as a contemporary, discriminating HIV-HRQL measure incorporating influential emotional dimensions such as stigma. Here we present the first *within-country* results of PROQOL-HIV using qualitative and quantitative data collected from a West Australian cohort who participated in the development and validation of PROQOL-HIV, and provide a comprehensive picture of HRQL in our setting.

**Methods:**

We carried out a secondary analysis of data from Australian patients who participated in the international study: 15 in-depth interviews were conducted and 102 HRQL surveys using the PROQOL-HIV instrument and a symptom questionnaire were administered. We employed qualitative methods to extract description from the interview data and linear regression for exploration of the composite and sub-scale scores derived from the survey.

**Results:**

Interviews revealed the long-standing difficulties of living with HIV, particularly in the domains of intimate relationships, perceived stigma, and chronic ill health. The novel PROQOL-HIV instrument discriminated impact of treatment via symptomatology, pill burden and treatment duration. Patients demonstrated lower HRQL if they were: newly diagnosed (p=0.001); naive to anti-retroviral treatment (p=0.009); reporting depression, unemployment or a high frequency of adverse symptoms, (all p<0.001). Total HRQL was notably reduced by perceived stigma with a third of surveyed patients reporting persistent fears of both disclosing their HIV status and infecting others.

**Conclusions:**

The analysis showed that psychological distress was a major influence on HRQL in our cohort. This was compounded in people with poor physical health which in turn was associated with unemployment and depression. People with HIV infection are living longer and residual side effects of the earlier regimens complicate current clinical management and affect their quality of life. However, even for the newly diagnosed exposed to less toxic regimens, HIV-related stigma exerts negative social and psychological effects. It is evident that context-specific interventions are required to address persistent distress related to stigma, reframe personal and public perceptions of HIV infection and ameliorate its disabling social and psychological effects.

## Background

Antiretroviral therapy (ART) has slowed progression of HIV infection to AIDS, and significantly reduced morbidity and mortality in people living with HIV (PLWH) who have access to treatment [1]. In addition, increased tolerability of ART [2], reduced pill burden and dosing frequency have been positive influences on health- related quality of life (HRQL) facilitated by medication adherence [3]. However, many PLWH who survived the early era of combination therapy experience residual disabling effects of the drugs such as: lipodystrophy [4], neuropathy [5], and persistent immunosuppression [6], which can impair quality of life. Comorbidities such as hepatitis C may add an additional physical and psychological burden [7-9]. Social circumstances such as unemployment and inadequate housing [10], and emotional stressors—often related to HIV-related stigma [11]—can also diminish HRQL [12]. A marked feature of HIV-related stigma is that PLWH are often attributed as agents responsible for the potentially contagious and fatal infection, and associated with practices marked as deviant by many societies [13]. Significantly, stigma can have an intrapersonal dimension when acceptance of society’s views leads to a form of self discrimination where one feels deserving of society’s disapprobation [13,14] and this internalised stigma appears predictive of significant outcomes for PLWH [15,16]. Notably, stigma is a complex social construct and it is likely to impact differentially according to an individual’s social context [17-19]. Evidently, these stressors continue to exert an influence on health-related quality of life (HRQL) [20] despite advances in clinical care.

Measures to assess the effect of drugs for the treatment of HIV on health status and wellbeing were introduced in 1991 [21] and while HRQL matters irrespective of treatment status—since the purpose of ART is to suppress the virus—it is necessary to ensure that HRQL is not compromised during this process. Accordingly, measures are commonly used to either detect changes in HRQL that may make drug or other new interventions more or less acceptable to patients and prescribers, or to indicate what other pressures, independent of treatment, may affect people living with HIV. A new Patient-Reported Outcomes (PRO) questionnaire to measure the HRQL in people living with HIV was recently developed and validated internationally with the participation of our centre [22,23]. In this paper, we juxtapose the hitherto unreported qualitative data and the discrete quantitative data obtained from the Australian participants. The aim of this study was to enhance understanding of the major factors impacting health-related quality of life in our cohort. By focusing on this local perspective, the work provides an explanatory rather than predictive approach to understanding the relationship between HRQL and physical, social and psychological distress, including HIV-related stigma, on a specific sample in a particular context.

## Methods

### Study design and setting

Semi-structured interviews with fifteen patients and survey data from 102 (out of 106 administered) respondents were analysed. It had been determined that fifteen interviews were sufficient for a within-country analysis based on the research of others into qualitative data saturation [24], and the sample size for a within-country analysis of the PROQOL-HIV questionnaire had been powered for 100 patients. This figure considered the number of conceptual dimensions needed to ensure a final factor model with reliable factor loadings in exploratory factor analysis [23,25]. Patients with HIV infection, over the age of 18 years and able to give informed consent, were eligible for inclusion in the study. Patients were recruited during scheduled clinic visits to the Immunology Clinic at Royal Perth Hospital, a tertiary ambulatory facility. The fifteen interviewees were informed about the study by the unit social worker, and patients subsequently contacted the researcher to arrange an appointment at an agreed venue. These participants were chosen to reflect a mix of age, gender and mode of HIV transmission. Recruitment for the survey phase was conducted at consecutive clinics from June to October 2008. Patients with sufficient time before their appointment with the physician were invited to complete the survey. Of 109 patients approached, two patients declined to participate and data from five surveys were not included in the final analysis because they were incomplete. The study was approved by the Royal Perth Hospital Ethics Committee (2007/115).

### Interview methods

Two trained interviewers conducted semi-directed interviews of 60 to 120 minutes duration which were recorded and transcribed verbatim. The interview guide comprised 107 questions of which the first five were open-ended seeking participants’ experiences of the impact of HIV on their daily lives and their quality of life in general. The latter 102 questions were categorised into topics of self and body care, daily activities, physical activities, health perception, energy/fatigue, cognitive functioning, social relationships, emotions and treatment. Participants were invited to qualify their responses. Hard copies of the transcripts were printed and patient interview data were examined by the two interviewers, independently, and several themes were identified and discussed. The transcripts were subsequently imported into the NVivo 8 qualitative software programme (QSR International Pty Ltd) where the verbatim was catalogued under theme headings called nodes. Analysis in NVivo facilitated the exploration of recurring themes and concepts, and drew attention to commonalities and variation between the verbatim of the interviewees.

### Questionnaires

The PROQOL-HIV questionnaire and a 31-item HIV symptom index [26] modified to include self-reported signs of fat redistribution and symptoms not found in the list, were administered concurrently. The questionnaires were completed in face-to-face communications to allow the participants the opportunity to ask questions and make comments during their completion.

The global PROQOL-HIV score, as used in analyses, is a composite measure based on 38-items, comprising eight sub-scales (factors) that encompass the following domains: Physical health and symptoms (PHS), emotional distress (ED), health concerns (HC), body change (BC), intimate relationships (IR), social relationships (SR), stigma (ST). The last sub-scale, treatment impact (TI) is omitted in a seven-factor score so that the questionnaire is equally applicable to patients irrespective of treatment status. The sub-scales were derived from summed Likert item values (range 0–4 points) expressed as a final score on a 0–100 scale with higher values indicating better HRQL; these were then averaged to arrive at the composite global score. The main properties, including the validity and reliability of the questionnaire and comparisons with other commonly used HRQL instruments, are described elsewhere [23], but briefly, the score’s reliability using Cronbach’s alpha was 0.936 (95% CI = 0.929-0.943); and the intra-class correlation coefficient (ICC) was based on an assessment of 34 patients for temporal stability and resulted in a score of 0.86 (95%, CI=0.701-0.959).

### Clinical characteristics

Plasma HIV RNA levels were determined using a polymerase chain reaction assay with a lower limit of detection of 40 copies/ml (Roche). The CD4+T cell lymphocyte count (mm^3^) was measured using the current standard flow cytometry assay (FACScanto™ flow cytometer, Becton Dickinson). Histories of HIV/AIDS diagnosis, co-morbidities and treatments were collected from the patients and verified using their medical records. Adherence to ART was measured as the number of self-reported missed doses in the two weeks preceding the study visit and was concordant with the standard clinic practice of recording level of adherence, longitudinally, for all PLWH attending the clinic [27].

### Analysis

Sociodemographic, biomedical and psychometric data obtained from the PROQOL participants were compared across treatment groups (no HIV therapy, or treatment based on a protease-inhibitor or a non-nucleoside reverse transcriptase inhibitor) using Fisher or Kruskal-Wallis tests as appropriate. Quantitative analysis of the PROQOL scores was exploratory, with an aim to identify correlates of HRQL and assess their relative association across contributing sub-scales. To this end, both the composite and sub-scale scores were analysed as dependent variables in a series of linear regression models which considered a suite of potential correlates as independent variables both univariately and in blocks according to type: sociodemographic, treatment-related, biological or clinical. Results of the initial univariate analyses are reported as mean (SE) changes in scores, and the block multivariate analyses as model R^2^ values which measure the proportion of the variation in scores explained by the variable sets considered as a whole. This approach was chosen to focus on relative impact according to correlate type, thereby lending itself to more meaningful interpretation in our context. Secondary multivariate analyses were then undertaken to further explore several effects highlighted in the univariate analyses, in particular to assess independence of specific noteworthy factors having a univariate p < 0.05. Analyses were undertaken using TIBCO Spotfire S+ 8.2 for Windows (TIBCO Software Inc., Palo Alto, CA).

### Results of interviews

#### Clinical and sociodemographic data

Most of the 15 interviewees were Caucasian men, three were SE Asian and two identified as being of Aboriginal ancestry. The median year of HIV diagnosis was 2001, and the mean (± SD) age was 41 yrs (±10.5), CD4 T-cell count 542 copies/ml (±332.2), and percent of lymphocyte count 25.5% (±9.7) (Table 1). Of the 3/15 patients who had a detectable viral load, two were not on current ART and one had a history of poor adherence. Symptoms were frequently reported (1–20 symptoms/31) at an average of eight symptoms per person. The most common were gastrointestinal (11 patients) followed by sleep difficulties (10), fatigue (10), and skin dry/itching (9). Eight patients smoked and eleven reported current use of alcohol. Seven lived alone, five in a couple, two with family members and two with children. Six patients were in paid employment and two were students. All completed school to at least age 15 and six were tertiary educated. Co-morbidities were described by 4/15 (26%): hepatitis C (HCV) (2 patients), pulmonary hypertension (1), Type 2 diabetes (1), and chronic back pain (1) and four (26%) reported current depression, two were treated. Three patients had lipodystrophy attributed to previous treatment with thymidine analogue nucleoside reverse transcriptase inhibitors.

**Table 1:** Characteristics of the 15 interviewees

**Patient**	**Age (yrs)**	**Gender m/f/o**	**Transmission mode**	**Year**	**CDC**	**CD4 (%)**	**Viral Load (log)**	HAART*	**Duration (mths)**
1	48	m	msm	1985	C	35	<40	3TC/AZT/ABC	109
2	31	m	msm	2004	A	30	<40	3TC/AZT/EFV	42
3	41	f	unknown	1987	B	22	<40	FTC/TDF/LPV/RTV	180
4	66	m	hetero	1997	C	20	<40	3TC/DDI/RTV/ATV	114
5	28	f	hetero	1994	C	<6%	4.8	no current HAART	156
6	36	m	msm	2003	A	28	1.84	3TC/AZT/NVP	33
7	55	m	msm	1988	A	34	<40	3TC/ ABC/ LPV/RTV	78
8	47	m	msm	2002	A	26	<40	3TC/ ABC/ LPV/RTV	36
9	45	m	msm	2001	C	28	<40	3TC/TDF/NVP	72
10	38	m	msm	2006	A	17	<40	3TC/AZT/EFV	9
11	26	f	hetero	1998	A	49	<40	3TC/AZT/EFV	72
12	32	f	IDU	2001	A	32	3.92	no current HAART	72
13	44	m	msm	1986	C	21	2.16	3TC/AZT/ABC	156
14	37	m	msm	2005	A	30	<40	3TC/ABC/RTV/ATV	26
15	42	m	msm	2002	C	20	<40	3TC/AZT/EFV	63

**Abbreviations: **msm = men who have sex with men, hetero= heterosexual, IDU = injecting drug use. HAART= highly active antiretroviral therapy; 3TC= lamivudine, FTC= emtricitabine, AZT= zidovudine, ABC = abacavir, TDF = tenofovir, DDI= didanosine, NVP= nevirapine, EFV= efavirenz, LPV=lopinavir, RTV= ritonavir, ATV= atazanavir; 8/13 patients on a BD regimen, mean number of pills = 3.4, median=3.

CDC = stage at first presentation.

#### Interview verbatim: aspects of living with HIV

Quotes illustrating four dimensions of living with HIV (Table 2) are presented: (1) emotional/social, (2) physical, (3) impact of treatment and (4) understanding of quality of life. Most patients perceived HRQL as having HIV or non-HIV influences. For some it involved feeling happy, and having supportive networks of family and/or friends, a satisfactory income and access to potent and effective treatments. The patients who experienced lower HRQL described co-morbidities, a higher frequency of stigma fears, self-reported adverse symptoms and were more likely to be on disability pensions.

**Table 2:** Interviews: Themes emerging from narratives (N=15)


**Emotional and social impact**	*The fact that (it’s, a) it’s a scary thing for people that don’t have it and a scary thing for people that do have it, it’s just a terrifying thing… *(woman, 28 yrs).
	*What disturbs me most about HIV is even though there is a lot of information out there, there are a lot of people that still are quite ignorant and they’re still quite fearful of HIV (man, 31 yrs).*
	*That my mother will find out…(man, 42 yrs).*
	*I am scared that I tell them and then it will be different. They would treat me differently (woman, 28 yrs).*
	*I’m afraid of dying…getting sick and not being able to take care of my children. I’m afraid of my children finding out like that’s probably my biggest fear in this world is when my children find out *(woman, 32 yrs).
	*Oh massively restricted in the sexual sense* (woman, 26 yrs).
	*The fear that something may happen to me and I’m in a car accident....and someone tries to help me and I’m bleeding profusely and I pass it on to them *(woman, 32 yrs).
	*Sometimes if I’m cooking… silly thoughts of cutting myself *(man, 37 yrs).
	*When they talk about people there is a disgust in the way they talk there’s disgust and it really deeply hurts because I have HIV and I don’t think that I’m disgusting *(woman, 28 yrs).
	*Sometimes it’s really difficult… like can be very isolating. Sometimes I feel like no one really understands and it’s very secretive as well like no one really knows, I don’t want people knowing *(woman 32 yrs).
	*…the friends that I did have put distance between us…. Separated themselves from me because it’s all got a bit hard or whatever *(man, 47 yrs).
	*That fear of possibly infecting him. I think the guilt eventually killed it *(man, 42 yrs).
**Physical impact**	*I went straight back to work as soon as I could- (work) was just a very lucky distraction to have *(man, 38 yrs).
	*I get tired very quick but don’t generally stop me doing anything I want to do…my problem is not wanting to do anything…it’s the motivation I don’t have *(woman, 41 yrs).
	*Tired and exhausted both *(man, 66 yrs).
	*I might have three or four bad days in a row and I have to ring up and take it off work and there’s no employer… they can’t put up with that for too long *(man, 47 yrs).
	*…any sort of activity that involves danger, all the risk of you know getting cut or something like that I just don’t do* (woman, 32 yrs).
**Treatment impact**	*I look at them making me sick, making me vomit like just want to vomit already just looking at them *(woman, 28 yrs).
	*The drugs, the drugs are great. I can’t complain I haven’t had any side effects *(man, 55 yrs).
	*None… problem is just taking them in front of people* (man, 37 yrs).
	*I never had a eight hour sleep… there was not such a thing as an eight hour sleep *(man, 44 yrs).
**Perceptions of quality of life**	*The way you do to get to do the things you like to do, like what kind of barriers do you face like - do you have the support networks and all that sort of stuff *(man, 66 yrs).
	*… means enjoying yourself, being happy… feeling good within yourself and about the people around you. I don’t have that any more *(man, 47 yrs).
	*I think within the realms of HIV itself, I would think that my health is good… um… but within the realm of the general population, I would say that my health is quite bad…* (man, 42 yrs).
	*Since my medication my life’s actually been enhanced* (man 37 yrs).
	*I’ve got the best drugs, good treatment at the hospital - I can still work *(man, 38 yrs).
	*It’s been up and down but it’s now it’s pretty good according to all the numbers and results and things, I’m pretty well normal… *(woman, 41 yrs).
	*I realised it’s all psychological really *(woman, 32 yrs).
	*I suppose for two thirds of my life, because … I have been living with HIV for a third of my life, I lived a quality of life where I didn’t have to think about everything I just did what I wanted to do basically within ones responsibilities and obligations. Now through HIV my quality of life has changed in that now everything I do has to be considered (man, 45 yrs).*

#### Emotional and social impact

Feelings of social stigma triggered by HIV were associated with fear and anxiety in 11 out of the 15 interviewees despite a degree of self-reported adjustment to living with HIV. A fear of transmitting HIV inadvertently or in a situation where one was powerless to protect others, for example, during a car accident, was intense for some individuals. A minor cut while preparing food resulted in interviewees starting the process again. One participant abandoned a profession in catering because of this concern. There was an aversion to disclosing HIV sero-status in any circumstance and a fear of being ‘outed’ by the appearance of ill health or being seen attending the hospital clinic. Anxiety about disclosure to employers, sexual partners and parents and, for two of the women, their children, was considered most stressful. Some perceived career options to be limited. Attitudes of others featured strongly; stigma related to disease but also sexual identity, for example a heterosexual man feared homophobia. HIV impacted on work opportunities for those with ill health and choice of work generally. Feelings of sadness, shame and inferiority were common. The greatest restrictions for over half of participants were around sexuality: reduced spontaneity and avoidance of sexual intercourse. The legal and moral requirement to disclose to prospective partners and, potentially, employers weighed heavily. Restrictions to travel opportunities were perceived, compounded by concerns over travelling with medication, disclosing HIV status on entry to some countries, and fear of becoming ill while holidaying. Some withdrew by degrees from social activities. The loneliness of chronic illness was described by one participant who perceived that friendships had fallen away since illness has altered his appearance and relationships with health care providers are tense because of his inability to ‘get well’ despite their ‘efforts’. However, approximately half of the sample expressed either acceptance or adjustment of their HIV serostatus and two reported receiving support following disclosure.

#### Physical impact

With regard to physical activity, some patients were limited by disability related to co-morbidity, whilst in others symptoms of pain and/or fatigue limited activity and affected motivation resulting in feelings of social isolation. Activity that might result in transmission of HIV was avoided by interviewees and some had given up pleasurable body contact sports and professions which they perceived might lead them to spill blood.

#### Impact of treatment

The interviewees who started ART pre-2000 described effects of pill burden, frequent dosing intervals and residual side effects. One developed immune restoration syndrome and a disfiguring skin condition. His experience of high-dose steroids and frequent changes of ART were debilitating and he felt unable to work or maintain an intimate relationship. Another interviewee had a long-standing history of non-adherence to ART and was hospitalised with severe immunodeficiency. This respondent took medication secretly, as did others. However, the majority viewed ART positively, as improving their health despite the effects of long-term toxicities.

### Results of the PROQOL-HIV survey questionnaire

#### Clinical and sociodemographic data

The data were collected from 102 patients (15% female) aged between 24 and 71 years. Socio-demographic characteristics can be found in Table 3. Transmission was mainly through sexual contact (94%) and nearly 40% of participants lived alone. All but two patients reached at least secondary school level, and 80% were employed. The most common co-morbidity was depression (24%) followed by HCV (17%), psychiatric disorder (5%), cardiovascular disease (3%) and hepatitis B (2%). Of the patients with depression, 77% were treated with antidepressants and 30% had hepatitis C. Most patients were treated with ART (87/102), two patients had stopped treatment, and 13 were ART naive. Amongst treated patients, 76% reported 100% adherence over the last two weeks, and of the 47% on a protease inhibitor (PI) based regimen 56% were taking ART once daily compared with 83% of those on a non-nucleoside reverse-transcriptase inhibitor (NNRTI) regimen. An undetectable viral load was recorded in 85% of patients and the average CD4 T-cell percentage was 26%.

**Table 3:** Characteristics of PROQOL participants

**Variables**	**ALL**	**Not on Rx**	**NNRTI**	**PI**	**p-value**
	**N = 102**	**N = 15**	**N = 46**	**N = 41**	
**Female**	15 (14.7%)	4 (26.7%)	7 (15.2%)	4 (9.8%)	p=0.3
**Age**	46 (37–53.8)	41 (32.5-49.5)	48 (37–56.5)	45 (40–53)	p=0.4
**Transmission**					p=0.08
**Heterosexual/other**	42 (41.2%)	10 (66.7%)	21 (45.7%)	11 (26.8%)	
**IDU**	6 (5.9%)	0 (0%)	3 (6.5%)	3 (7.3%)	
**MSM**	54 (52.9%)	5 (33.3%)	22 (47.8%)	27 (65.9%)	
**Married**	34 (33.3%)	5 (33.3%)	17 (37%)	12 (29.3%)	p=0.7
**Caucasian**	80 (78.4%)	9 (60%)	37 (80.4%)	34 (82.9%)	p=0.2
**Living alone**	40 (39.2%)	8 (53.3%)	16 (34.8%)	16 (39%)	p=0.5
**Post-secondary**	37 (36.3%)	6 (40%)	17 (37%)	14 (34.1%)	p=0.9
**Unemployed/sickness benefits**	18 (17.6%)	2 (13.3%)	3 (6.5%)	13 (31.7%)	p=0.007
**Smoker ≥ 2 cigarettes/day**	41 (40.2%)	4 (26.7%)	19 (41.3%)	18 (43.9%)	p=0.0002
**Alcohol ≥ 2 glasses/day**	15 (14.7%)	1 (6.7%)	7 (15.2%)	7 (17.1%)	p=0.5
**Diagnosed < 2 years**	16 (15.7%)	8 (53.3%)	6 (13%)	2 (4.9%)	p=0.7
**Time since diagnosis (years)**	7.3 (2.9-15.6)	1.5 (0.8-3.8)	6.8 (3.2-11.9)	13.2 (6.2-18.9)	p<0.0001
**Body mass index**	24.3 (22–28)	23.6 (22–28)	25.4 (23–28)	23.9 (22–27)	p=0.5
**CD4 T cells**	530 (376–733)	546 (512–695)	591 (386–853)	455 (323–638)	p=0.1
**Undetectable VL**	77 (75.5%)	2 (13.3%)	43 (93.5%)	32 (78%)	p<0.0001
**Time on ART (years)**	4.2 (0.8-11.8)	-	4.4 (2.1-9.9)	10.7 (3.2-13.6)	p<0.0001
**On non-ART medication**	38 (37.3%)	3 (20%)	15 (32.6%)	20 (48.8%)	p=0.1
**ART pill burden (tablets/day)**	3 (2–5)	-	2.5 (2–3)	4 (3–5)	p<0.0001
**Non-adherent to ART**	18 (17.6%)	-	7 (15.2%)	11 (26.8%)	p=0.2
**Depressive**	26 (25.5%)	3 (20%)	8 (17.4%)	15 (36.6%)	p=0.1
**Other comorbidity**	56 (54.9%)	8 (53.3%)	20 (43.5%)	28 (68.3%)	p=0.07
**Sexual dysfunction**	25 (24.5%)	4 (26.7%)	9 (19.6%)	12 (29.3%)	p=0.5
**Number symptoms (total)**	5 (2–10)	4 (1–10.5)	5 (1–8)	8 (5–12)	p=0.01

For categorical variables, values are N(%) and the p-value corresponds to a Fisher exact test of equal proportions across treatment groups; for continuous variables, values are median (interquartile range) with the p-values reflecting differences across the treatment groups as assessed by a Kruskal-Wallis test.

### Assessment of HRQL

Quality of life outcomes were assessed in terms of both the global PROQOL score and the 8-subscale scores. The global 7-factor score had an observed mean (SE) of 60.62 (2.01), with mean subscale scores ranging from 43.32 (ST) to 72.55 (SR). Restricting to the treated patients only, scores were slightly higher across all domains except the physical health score, with the highest subscale being that measuring treatment impact. Inclusion of this latter domain resulted in an increase of the global mean from 61.66 (2.21) for the 7-factor score to 64.07 (2.06) for the 8-factor score.

Univariate regression analyses of demographic associations with the PROQOL global and subscale scores are presented in Table 4, with covariates grouped according to indication of sociodemographic, treatment, biological and clinical factors. As a whole, sociodemographic factors had a notable impact across all sub-scales. Professional activity was particularly discerning, with patients unemployed or on sickness benefits reporting consistently lower HRQL across the domains. Other factors were more domain-specific. Those living alone were significantly more impacted by difficulties with intimate relationships compared with those living with others (p = 0.006), whereas older age was associated with higher HRQL scores in this domain. Worry over HIV and other health outcomes, for example test results and catching other infections, was less common in Caucasians (p = 0.003) and older patients (p = 0.02), However, those recently diagnosed (<2 years) were more likely to express health concerns (p = 0.001) and, with lower scores in the stigma domain, indicate fear of disclosing their HIV status and infecting others (p = 0.02).

**Table 4:** **Linear regression estimates of univariate predictors of mean (SE) increase/reduction (+/-) in PROQOL subscale and global scores (body of table), together with the percent of the score variances explained by fitting joint models of covariate blocks as grouped according to indication of sociodemographic, treatment, biomedical or clinical factors (R**^**2**^**×100)**

	**Emotional**	**Health**	**Social**	**Intimate**	**Stigma**	**Body**	**Phys. Health & Symptoms**	**Treatment Impact^**	**Global**	
	**Distress**	**Concerns**	**Relationships**	**Relationships**		**Changes**				
									**7-factor^**	**8-factor**
**All patients**										
**Mean (SE)**	**64.89 (2.63)**	**54.90 (2.64)**	**72.55 (2.58)**	**51.63 (2.98)**	**43.32 (3.10)**	**66.48 (2.58)**	**71.36 (2.14)**	**-**	**60.62 (2.01)**	**-**
**Treated patients**										
**Mean (SE)**	**65.16 (2.88)**	**57.69 (2.80)**	**73.41 (2.79)**	**52.01 (3.17)**	**46.66 (3.40)**	**66.52 (2.73)**	**71.05 (2.27)**	**81.08 (1.63)**	**61.66 (2.21)**	**64.07 (2.06)**
**Sociodemographic factors**										
Male gender	+8.18 (7.43)	+13.56 (7.36)	+2.01 (7.33)	-9.81 (8.41)	+9.77 (8.71)	+4.18 (7.32)	+3.09 (6.05)	+7.36 (4.86)	+4.29 (5.67)	+4.50 (6.18)
Transmission (ref: heterosexual/unknown)										
IDU	+11.61 (11.65)	+14.29 (11.65)	-0.89 (11.49)	+2.28 (13.14)	+27.39 (13.43)*	+1.64 (11.49)	+1.29 (9.48)	+3.46 (6.77)	+8.51 (8.89)	+7.60 (8.57)
MSM	+3.51 (5.49)	+3.17 (5.49)	-2.28 (5.42)	-8.60 (6.19)	+8.41 (6.36)	+2.56 (5.42)	-3.01 (4.47)	+4.34 (3.46)	+0.82 (4.21)	+2.20 (4.41)
Caucasian race	+11.38 (6.33)	+18.93 (6.16)**†**	+4.84 (6.29)	-2.75 (7.29)	+5.98 (7.52)	+10.87 (6.22)	+4.29 (5.20)	+11.34 (4.04)**†**	+7.52 (4.84)	+9.16 (5.23)
Age >45 years	+9.04 (5.22)	+12.21 (5.16)*	+8.24 (5.13)	+12.32 (5.87)*	+11.00 (6.13)	+5.18 (5.17)	+6.27 (4.25)	+9.25 (3.12)**†**	+9.02 (3.95)*	+9.23 (4.02)*
Living alone	-0.33 (5.42)	-2.15 (5.42)	-11.91 (5.18)*	-16.74 (5.91)**†**	-6.87 (6.36)	-2.18 (5.32)	-6.44 (4.35)	+2.82 (3.38)	-7.05 (4.1)	-4.23 (4.29)
Diagnosed <2 years	-0.98 (7.27)	-22.96 (6.91)**†**	-2.65 (7.13)	-3.79 (8.24)	-18.98 (8.32)*	+4.54 (7.13)	+6.54 (5.86)	-7.04 (5.61)	-5.34 (5.52)	-3.04 (7.12)
Unemployed or	-23.14 (6.54)**‡**	-5.95 (6.92)	-20.63 (6.49)**†**	-16.88 (7.69)*	-2.01 (8.14)	-25.92 (6.3)**‡**	-27.45 (4.91)**‡**	-10.32 (4.07)*	-17.32 (4.99)**‡**	-16.34 (5.01)**†**
sickness benefits										
Post-secondary	+8.44 (5.44)	+3.18 (5.5)	-0.4 (5.4)	-0.8 (6.24)	-0.65 (6.47)	+3.03 (5.39)	+8.22 (4.39)	-0.92 (3.42)	+3.2 (4.19)	+2.17 (4.31)
education										
Substance use over previous 2 weeks										
Tobacco	+1.1 (5.4)	+1.49 (5.4)	-8.65 (5.22)	+6.95 (6.08)	+1.75 (6.37)	-4.62 (5.28)	-7.68 (4.31)	-0.81 (3.31)	-1.69 (4.13)	-1.09 (4.2)
Alcohol	-3.29 (7.46)	+10.37 (7.41)	-0.06 (7.33)	+0.04 (8.47)	-0.96 (8.76)	+4.12 (7.32)	+1.92 (6.06)	+3.18 (4.44)	+1.88 (5.69)	+3.57 (5.6)
**Variance explained by sociodemographic factors**										
**(proportion)**	**18.01%**	**22.49%**	**19.64%**	**20.7%**	**18.74%**	**20.69%**	**32.63%**	**20.6%**	**22.61%**	**21.22%**
**Treatment factors**										
On ART	+1.82 (7.47)	+18.94 (7.24)*	+5.92 (7.31)	+2.57 (8.47)	+22.49 (8.46)**†**	+0.27 (7.33)	-2.08 (6.06)	-	+7.00 (5.65)	-
Current ART (ref: NNRTI)										
none	- 3.79 (7.96)	-19.27 (7.74)*	-7.50 (7.80)	-6.44 (8.98)	-15.00 (8.80)	-2.09 (7.82)	-2.99 (6.30)	-	-7.95 (6.04)	-
PI	-4.16 (5.75)	-0.70 (5.59)	-3.35 (5.63)	-8.23 (6.48)	+15.71 (6.37)*	-3.86 (5.65)	-10.76 (4.55)*	-9.93 (3.10)**†**	-1.99 (4.37)	-2.97 (4.13)
Time on HAART	+0.23 (0.5)	+1.38 (0.47)**†**	-0.07 (0.49)	-0.04 (0.56)	+1.44 (0.57)*	-0 (0.48)	-0.12 (0.4)	+0.36 (0.28)	+0.43 (0.38)	+0.42 (0.36)
(per year) ^**^**^										
ART pill burden	-2.78 (2)	-0.68 (1.96)	-2.06 (1.94)	-3.74 (2.18)	+3.06 (2.36)	-2.12 (1.9)	-3.06 (1.55)	-3.36 (1.08)**†**	-1.56 (1.54)	-1.78 (1.43)
(per tablet/day) ^**^**^										
ART non-adherent^**^**^	-2.48 (7.16)	+2.13 (6.96)	-3.26 (6.93)	-7.21 (7.84)	-2.8 (8.4)	-1.57 (6.79)	-6.58 (5.58)	-9.22 (3.91)*	-2.95 (5.46)	-3.72 (5.07)
On non-ART	-8.00 (5.41)	-2.57 (5.47)	-6.05 (5.34)	-6.45 (6.17)	+8.08 (6.38)	-11.07 (5.26)*	-12.39 (4.26)**†**	-1.19 (3.34)	-5.35 (4.14)	-4.61 (4.18)
medication										
**Variance explained by treatment factors**										
**(proportion)**	**8.33%**	**20.27%**	**4.02%**	**5.39%**	**17.01%**	**6.51%**	**14.28%**	**26.55%**	**10.18%**	**10.18%**
**Biological factors**										
Body mass index										
(per unit)	-0.01 (0.58)	-0.35 (0.58)	-0.29 (0.57)	+0.20 (0.66)	+0.02 (0.68)	+0.08 (0.57)	-0.18 (0.47)	+0.16 (0.35)	-0.07 (0.44)	-0.01 (0.44)
CD4 count (per 100										
T cells)	+0.25 (0.82)	+1.50 (0.81)	+0.30 (0.81)	+1.90 (0.91)*	+0.22 (0.97)	+0.80 (0.80)	+0.42 (0.66)	+0.88 (0.47)	+0.81 (0.62)	+0.84 (0.60)
Detectable viral										
load	-9.13 (6.08)	-17.09 (5.92)**†**	-10 (5.95)	-6.36 (6.94)	-13.05 (7.10)	-5.94 (6.01)	-0.87 (4.99)	-10.52 (4.61)*	-8.79 (4.6)	-10.96 (5.86)
**Variance explained by biological factors**										
**(proportion)**	**2.99%**	**11.15%**	**3.31%**	**4.57%**	**3.47%**	**2.07%**	**2.49%**	**10.00%**	**4.80%**	**5.51%**
**Clinical factors**										
Depressive	-27.84 (5.41)**‡**	-11.26 (5.95)	-19.64 (5.63)**‡**	-20.76 (6.51)**†**	+4.91 (7.12)	-17.22 (5.69)**†**	-24.26 (4.27)**‡**	-6.49 (3.67)	-16.48 (4.29)**‡**	-16.42 (4.29)**‡**
Other comorbidity	-10.5 (5.21)*	+0.27 (5.32)	-3.97 (5.2)	-3.95 (6.02)	+9.49 (6.2)	-7.59 (5.16)	-8.61 (4.23)*	-3.35 (3.27)	-3.35 (4.06)	-4.35 (4.14)
Symptoms										
Sexual dysfunction	-26.02 (5.57)**‡**	-22.06 (5.75)**‡**	-6.69 (6.00)	-23.14 (6.58)**‡**	-12.38 (7.11)	-14.88 (5.85)*	-23.6 (4.40)**‡**	-7.86 (3.73)*	-18.29 (4.31)**‡**	-16.03 (4.49)**‡**
Gastrointestinal	-6.73 (1.53)**‡**	-3.99 (1.63)*	-4.2 (1.59)**†**	-5.38 (1.82)**†**	-0.31 (1.97)	-5.63 (1.54)**‡**	-8.57 (1.05)**‡**	-3.3 (0.94)**‡**	-4.95 (1.18)**‡**	-5.06 (1.15)**‡**
(per number)										
Malaise (per	-8.11 (1.58)**‡**	-4.75 (1.71)**†**	-6.44 (1.62)**‡**	-4.28 (1.97)*	+0.17 (2.09)	-8.29 (1.53)**‡**	-9.18 (1.11)**‡**	-2.21 (1.12)	-5.95 (1.22)**‡**	-5.84 (1.3)**‡**
number)										
Morphological	-4.71 (1.36)**‡**	-2.2 (1.42)	-2.75 (1.38)*	-5.29 (1.54)**‡**	-1.04 (1.68)	-4.81 (1.33)**‡**	-4.97 (1.05)**‡**	-1.76 (0.84)*	-3.65 (1.03)**‡**	-3.66 (1.01)**‡**
(per number)										
Other (per number)	-8.39 (2.5)**†**	-4.6 (2.6)	-5.32 (2.53)*	-4.34 (2.96)	-0.8 (3.15)	-11.06 (2.34)**‡**	-9.17 (1.93)**‡**	-2.48 (1.58)	-6.71 (1.93)**‡**	-7.01 (1.91)**‡**
**Variance explained by clinical factors**										
**(proportion)**	**37.25%**	**19.37%**	**19.68%**	**21.82%**	**6.7%**	**29.97%**	**62.04%**	**14.2%**	**31.92%**	**33.31%**
**Variance explained by all factors**										
**(proportion)**	**63.64%**	**49.81%**	**37.24%**	**46.31%**	**33.07%**	**41.26%**	**75.36%**	**47.83%**	**53.01%**	**55.4%**

**^**Analysis restricted to treated patients only.

P-values: **‡** P<0.001; **†** 0.001≤P<0.01; * 0.01**≤** P<0.05.

Amongst patients receiving treatment, class of ART and adherence were independently associated with the domain capturing treatment impact; lower scores were observed amongst those on a protease inhibitor and those reporting less than 100% adherence (p = 0.005 and p = 0.048, respectively, in a joint model). Treatment-related factors impacted on other subscales as well. Patients on a protease inhibitor or taking medication for non-HIV comorbidities also recorded lower scores pertaining to physical health and symptoms. Furthermore, duration of ART, in particular, was positively associated with higher scores in both the health concern and stigma domains (p < 0.007), indicating an attenuation of anxiety over time. Accordingly, those receiving protease inhibitor-based ART who on average had been on therapy for 3 years longer than patients receiving NNRTI-based therapy, appeared less affected by issues of stigmatization.

**Figure 1:**
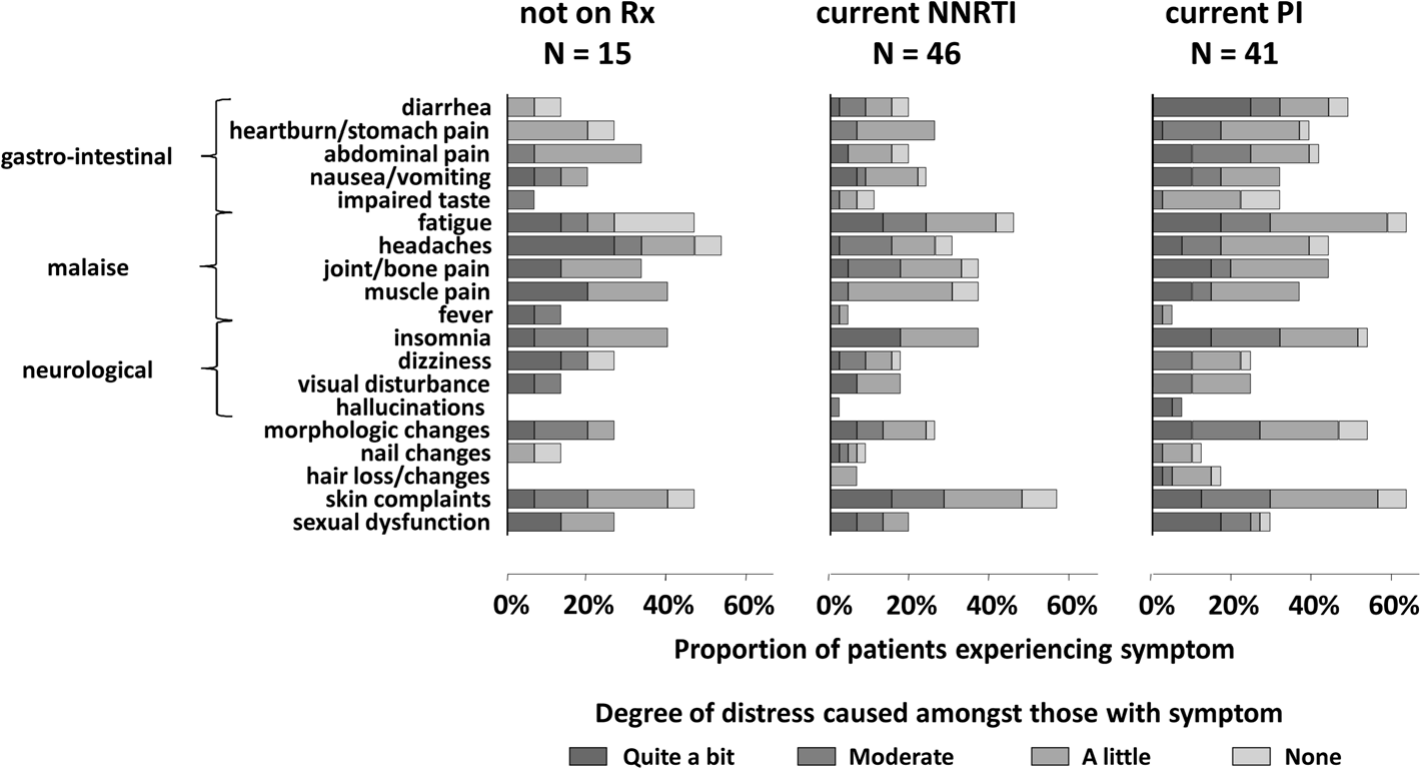
Patient reported symptoms and associated distress.

Negatively impacting on HRQL across many of the subscale scores were clinical factors: suffering from depression, another comorbidity, sexual dysfunction and frequency of experienced symptoms. The number of symptoms, in particular, was highly predictive of reduced HRQL across all domains except stigma. Symptoms were more prevalent amongst patients receiving PI regimens (mean (SE) number = 8 (0.85)) compared with those on NNRTI regimens (5.48 (0.80), p = 0.005, Mann–Whitney test) but were not confined to patients receiving ART (Figure 1). Symptoms of malaise, particularly, were common across all three groups, as were insomnia and skin problems.

Consistent with the observed impact of clinical factors across most of the sub-scales, lower global PROQOL-HIV scores were strongly associated with occurrence of symptoms, depression or other comorbidities (Table 4). When considered together in multivariable models these clinical factors explained over 30% of the variability in both the 7-factor and 8-factor global scores, contrasting with about 20% for the sociodemographic factors alone, 10% for the treatment factors and about 5% for the biological parameters. However, due to a lack of independence of the contributing factors their combined impact is not additive, and only a total of 55% of the PROQOL-HIV score variation could be explained by the complete covariate set.

Scores pertaining to stigma as captured by the PROQOL instrument were lower, on average, than all other domains and notable for lack of significant association with any of the clinical factors considered. This subscale is comprised of items pertaining to fear of disclosure of HIV status and transmitting the infection and there was a strong correlation between having a high frequency of these two fears (p = 0.0001), with 33% of participants reporting that fears of both disclosure and infecting others were often/always on their mind. These patients had consistently lower scores spread across subscales other than the stigma domain, particularly in the areas of emotional distress (p = 0.02), intimate relations (p = 0.0007), social relationships (p = 0.04) and health concerns (p < 0.0001). Whilst there appeared to be some attenuation of stigmatization with time, a substantial proportion of patients with these issues frequently on their mind had been on treatment for some time: 50% for at least 3 years and 25% for more than 7 years.

## Discussion

We have presented a thorough analysis of observational HRQL data in a single cohort using established qualitative methods and the new psychometric instrument, PROQOL-HIV. This is the first comprehensive study of HRQL in Western Australia since the HIV epidemic began thirty years ago. The results from the survey, supported by the interview material, demonstrate that HIV influences HRQL across the spectrum of biological, social and psychological domains that comprise the complex continuum of measures of health [28]. In particular, people reporting: unemployment, depression, and a higher frequency of symptoms, particularly those impacting negatively on sexual expression, scored a poorer quality of life overall, independently of other factors and regardless of ART status. Interview respondents struggling with romantic relationships described feelings of loss around cessation of sexual practices relinquished to prevent transmission; opportunities to engage in sexual activity without the burden of disclosure; fear of rejection and the potential for transmission despite use of safer sex strategies. Accordingly, the novel instrument has captured a dimension of HIV-related stigma, by way of an individual’s fear of disclosing their HIV serostatus and/or transmitting the infection that clearly results in emotional distress. Therefore, although the HIV/AIDS-Targeted Quality of Life Instrument (HAT-QOL) has a dimension for disclosure concerns [29], the PROQOL–HIV questionnaire goes a step further and juxtaposes anxiety about transmitting the infection with fear of disclosure.

In concordance with Lee’s research [30], the survey showed that feelings of stigma were heightened in those more recently diagnosed in contrast with those who had a longer history of HIV infection. These included the small number of patients infected via IDU who had a relatively long time since diagnosis (mean=12 years) and might be expected to experience compound stigma related to their membership of another marginalised group [31]. However, more than half of survey respondents expressing stigma concerns had been on therapy for over three years; and the interviews highlighted persistent concern that HIV-related stigma negatively affected relationships and employment opportunities, consistent with other studies [11,12,32,33]. Courtenay-Quirk and colleagues [34] found that avoidant coping strategies, anxiety, loneliness, depressive symptoms, and suicidal ideation were associated with HIV-related stigma in a community of HIV-positive men; and Holzemer [35] showed that stigma had a negative effect on quality of life independently of HIV-related symptoms and severity of illness. More recently Hutton and others [36], using the Personal Well Being Index [37] reported that stigma impacted negatively on subjective well-being in PLWH in Australia and the USA by way of perceived unsupportive (hurtful) social interactions.

While HIV-related stigma has been associated with depression [38], and specific concerns such as serostatus disclosure fears and transmission anxiety have been reported [11,30,39], we could not find evidence in the literature suggesting that transmission anxiety *per se* is a specific stressor in HIV-related depression and contagion fears appear more commonly addressed in uninfected individuals [40,41]. Cognitive behavioural interventions have been trialed to decrease HIV-related stigma and a recent study reported that an intervention improving personal control via a sense of mastery and increased social support may be beneficial in reducing stigma in people with depressive symptoms [38,40].

It was somewhat surprising then that reported depression did not correlate with the stigma domain in our study, and it is concerning that the fear of infecting others, which in some individuals resulted in avoidant behaviour out of proportion to risk, may not be uncovered in the course of clinical consultations. Since stigma may be nuanced by its various associations with sex, gender, death and ethnicity [31] and deeply internalised [13] it could be examined by assessing the limitations that people place upon their lives as a result of the anxiety.

A number of demographic factors contributed to a diminished perception of HRQL among the respondents to the interviews and the questionnaires. Notably, people living alone cited restricted intimate and/or social relationships, although whether this was as a result of self withdrawal or inhibition with regard to disclosing HIV status, or some other reason, is not clear. Older age and longer duration of HIV were associated with an improvement in HRQL, as observed by others [42]. In particular, older Caucasians were less troubled by health concerns related to regular CD4 and viral load monitoring and progression of the disease, perhaps reflecting their adjustment to diagnosis, better knowledge of the disease and/or greater confidence in the treatment, reinforced by successful treatment outcomes. However, unemployment and disability resulted in diminished HRQL regardless of age and the interviews revealed a picture of social isolation and physical discomfort not necessarily directly attributable to HIV disease.

People on PIs reported more symptoms, especially gastrointestinal, and more tablets overall compared with those on NNRTI regimens. This translated into greater treatment impact, but not into reduced HRQL overall, most likely because of improvements in overall physical health and perhaps psychosocial adjustments associated with the longer average duration of ART and time since diagnosis. The level of adherence in our patients was higher than in the other cohorts in the international study [23], perhaps reflecting once daily dosing, but we did not find a direct relationship between adherence and global HRQL. However, non-adherence to ART remained predictive of lower treatment impact scores over and above treatment choice and viral load. This finding suggests that the benefits of treatment were not perceived by non-adherent participants.

The study had been sufficiently powered to show significant site-specific effects of covariates on HRQL domains. However, the fact that the combined covariate sets explained only 55% in the PROQOL-HIV score variation suggests the total score may incorporate facets additional to quality of life specifically related to HIV, and is assessing additional information from a patient perspective that cannot be adequately inferred from the usual sociodemographic or biological variables. This finding concurs with Wilson’s [43] conceptual model suggesting that *total* HRQL is substantially impacted by some hard-to-measure factors relating to personality, which may contribute to resilience and coping.

There were limitations to our study. Cross-sectional design is less robust than longitudinal measurement where responses are measured over a period of time; however the qualitative component strengthens the internal validity of the study. The closed-ended format of the questionnaires did not accommodate explanations, but the questions were derived from the themes gathered in the interviews where patients qualified their responses to semi-directive questions freely.

The development of PROQOL-HIV, has allowed the measurement of dimensions not assessed in the past. Application in our local setting has demonstrated that the instrument will provide a useful tool in cohort analysis to assess health-related quality of life in general, and those that result from treatment interventions in particular. Inclusion of a stigma domain adds further utility since it is evident that stigma is a persistent feature of HIV infection and may result in emotional harm, particularly in those less resilient. The multiple nuances of stigma should be disentangled in future research in order to develop suitable interventions. In conclusion, disease-specific HRQL instruments can bring additional information to the classical criteria for evaluating clinical outcomes and should be part of studies evaluating health policy and treatment strategies [44].

The study was conducted at Royal Perth Hospital in Western Australia and was supported by the Assistance Publique–Hopitaux de Paris (AP-HP), GILEAD Sciences, and Sidaction.

## Competing interests

The authors declare that they have no competing interests.

## Authors’ contributions

SH managed the Australian arm of the PROQOL-HIV study, recruited patients, conducted interviews, entered data, carried out analysis of the qualitative data and wrote the manuscript; EMcK conducted statistical analyses and assisted in writing the manuscript; NH recruited patients, conducted interviews, analysed the qualitative data and reviewed the manuscript for critical content; CL conducted statistical analyses and reviewed the manuscript for critical content; SM, DN, and OC reviewed the manuscript for critical content; MD conceived the study, directed the design and reviewed the manuscript for critical content. All authors read and approved the final manuscript.
